# Monotonic Tension-Torsion Experiments and FE Modeling on Notched Specimens Produced by SLM Technology from SS316L

**DOI:** 10.3390/ma14010033

**Published:** 2020-12-23

**Authors:** Michal Kořínek, Radim Halama, František Fojtík, Marek Pagáč, Jiří Krček, David Krzikalla, Radim Kocich, Lenka Kunčická

**Affiliations:** 1 Department of Applied Mechanics, Faculty of Mechanical Engineering, VSB-Technical University of Ostrava, 17. listopadu 2172/15, 708 00 Ostrava, Czech Republic; radim.halama@vsb.cz (R.H.); frantisek.fojtik@vsb.cz (F.F.); david.krzikalla@vsb.cz (D.K.); 2 Department of Machining, Assembly and Engineering Metrology, Faculty of Mechanical Engineering, VSB-Technical University of Ostrava, 17. listopadu 2172/15, 708 00 Ostrava, Czech Republic; marek.pagac@vsb.cz; 3 Department of Mathematics and Descriptive Geometry, Faculty of Mechanical Engineering, VSB-Technical University of Ostrava, 17. listopadu 2172/15, 708 00 Ostrava, Czech Republic; jiri.krcek@vsb.cz; 4 Department of Materials Forming, Faculty of Materials Science and Technology, VSB-Technical University of Ostrava, 17. listopadu 2172/15, 708 00 Ostrava, Czech Republic; radim.kocich@vsb.cz; 5 Institute of Physics of Materials, Academy of Science of Czech Republic, Žižkova 513/22, 616 62 Brno, Czech Republic; kuncicka@ipm.cz

**Keywords:** stainless steel 316L, additive manufacturing, multiaxial loading, plasticity, digital image correlation method, hill yield criterion, isotropic hardening, finite element method (FEM)

## Abstract

The aim of this work was to monitor the mechanical behavior of 316L stainless steel produced by 3D printing in the vertical direction. The material was tested in the “as printed” state. Digital Image Correlation measurements were used for 4 types of notched specimens. The behavior of these specimens under monotonic loading was investigated in two loading paths: tension and torsion. Based on the experimental data, two yield criteria were used in the finite element analyses. Von Mises criterion and Hill criterion were applied, together with the nonlinear isotropic hardening rule of Voce. Subsequently, the load-deformation responses of simulations and experiments were compared. Results of the Hill criterion show better correlation with experimental data. The numerical study shows that taking into account the difference in yield stress in the horizontal direction of printing plays a crucial role for modeling of notched geometries loaded in the vertical direction of printing. Ductility of 3D printed specimens in the “as printed” state is also compared with 3D printed machined specimens and specimens produced by conventional methods. “As printed” specimens have 2/3 lower ductility than specimens produced by a conventional production method. Machining of “as printed” specimens does not affect the yield stress, but a significant reduction of ductility was observed due to microcracks arising from the pores as a microscopic surface study showed.

## 1. Introduction

The austenitic stainless steel AISI 316L is one of the most utilized constructional materials for various parts in the power industry and beyond. It was investigated in the conventional wrought state [1], while it has been loaded in tension, torsion, and even combinations of both. Nevertheless, it is increasingly utilized in the additively manufactured form [2], as it opens new possibilities. This was the motivation for the conference paper [3], which is the forerunner of this further extended paper. Stainless steel 316L (SS316L) may be optimized and applied in an organic shape or can even serve as a custom made part or machine element utilized in the repair or reconstruction of a structure, where commercial products are not available or are hardly producible by conventional manufacturing, such as machining. Various process parameters used during the additive manufacturing of SS316L have been examined [4,5]. One of the important outputs are the mechanical properties [6,7] or porosity [8]. The building direction also plays a vital role [9], and the final surface roughness is of particular interest [10]. It is well known that printing the layer by layer and natural cooling from the bottom directly lead to the formation of residual stresses in the material. The influence of the scanning strategy on the resulting residual stresses was also intensively studied for SS316L in recent years [11,12,13]. There are many studies available, which are focused on mechanical properties research, including anisotropy induced by the selective laser melting (SLM) process in the literature. However, there is still missing information about the plasticity of SS316L prepared by SLM under various multiaxial stress states. To simulate critical loading states and nonstandard events in technical practice, it is important to realize the necessary monotonic experiments using biaxial testing machines. This is an important motivation for research in the field of multiaxial plasticity.

This paper presents new results for the deformation response obtained during monotonic multiaxial loading of specimens made from SS316L, produced by SLM technology in the “as printed” state. Almost all specimens were printed in the vertical direction to prevent the effect of residual stresses. Due to the character of the specimens that contain notches, the digital image correlation (DIC) method was used. The DIC method is a progressive optical-numerical method suitable for 3D analysis of structural components under uniaxial and multiaxial loading in the full-field [14,15]. Averaged characteristics gained in this experimental study with DIC measurements were used for the validation of a numerical model based on the finite element method (FEM).

## 2. Materials and Methods

In preprocessing phase, the specimens were created with SOLIDWORKS 2019 (Dassult Systemes SoliDWorks, France, version2019). Subsequently, Powder Bed Fusion 3D printing technology, selective laser melting, was used for the production using a 3D printer Renishaw AM400 (Renishaw, New Mills, UK, 2016), and the material was atomized SS316L powder. This is an additive manufacturing technology, where the laser scans and selectively melts the atomized metal powder particles, bonding them together and building a model layer-by-layer [16,17]. In the beginning of the process, the building chamber was filled with inert gas argon to minimize the oxidation of the metal powder. The layer thickness was set to 50 μm, and the chessboard strategy was used. The strategy translates by 5 mm in the horizontal direction *X* and *Y* and rotates for the optimum homogeneous distribution of stress [18]. 

**Figure 1:**
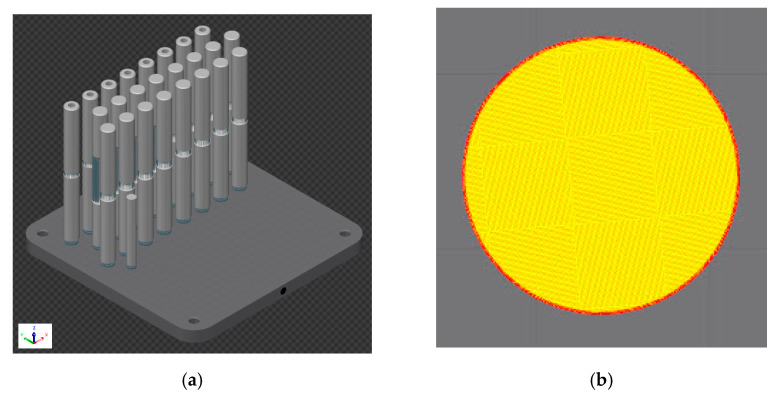
Part orientation and position in chamber: (**a**) 3D printing preview; (**b**) chessboard strategy preview in the cross-section.

In general, each specimen always had a different percentage and volume of porosity. The porosity varied with various parameters, such as the number of layers laid and the printing time. The study [19] dealt with the optimization of those parameters, and the production parameters from this study were used to produce the specimens in our study. These production parameters guaranteed a porosity of more than 99.9%. Other 3D printing parameters are shown in Table 1 [19,20] (QuantAM, SW made by company Renishaw) [21]. Building time was 76 h. The part orientation and the position in the chamber, the 3D printing preview, and the chessboard strategy preview in the cross-section are presented in Figure 1. The specimens were separated from the base plate with a band saw. Due to the printing direction, the effect of residual stresses was not expected.

**Figure 2:**
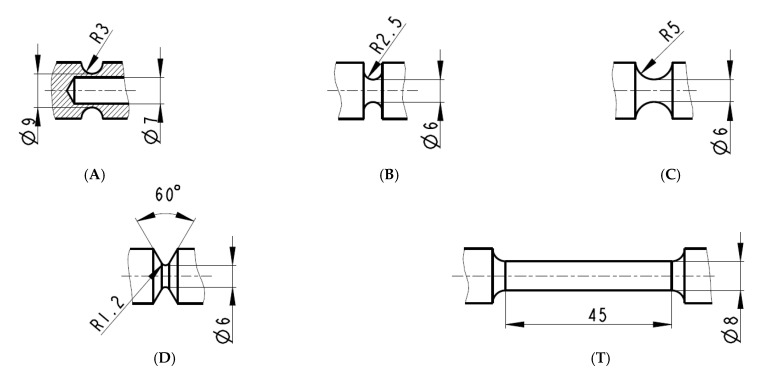
Specimens geometry: The tubular specimen with a notch for multiaxial loading (**A**), solid specimens with a notches for axial loading (**B**–**D**), and unnotched specimen for the standard tensile test (**T**).

The specimens were not further machined (outer surface) or heat treated (in the “as printed” state), therefore had naturally high surface roughness. The geometry of the notches considered in this study is shown in Figure 2. Only one type of specimenswas tubular, other were solid bodies. Tubular (specimens *A*) had to be drilled to the required internal diameter. Each specimen was 160 mm long, and the outer diameter was 15 mm. In addition, the standard tensile test was performed. The solid specimens were loaded only in tension. The tubes were subjected to two different loading modes: pure tension and pure torsion. Each mechanical test with pure axial loading was repeated four times. The torsion test was repeated only two times. The testing machine, LabControl 100 kN/1000 Nm (Opava, Czech Republic), was used. Multiaxial tests were done under deformation control with a 2 mm per min elongation rate for tension and under 0.157 radians per min twist rate for torsion. All tests were conducted at ambient temperature. The results of the tests were evaluated in the form of force (torque) vs. elongation (twist) diagrams.

DIC measurements were used to monitor the deformation. This method is characterized by the creation of a light area with dark points, also known as a pattern. Two optical sensors were used for this measurement to get 3D strain data. The sensors were high resolution cameras. The principle of DIC measurement was for two images of the specimen to be compared at different loading states using the appropriate facet size in pixels. Simultaneously, the images from both cameras were correlated in real-time time to get the contours of the specimen’s surface. Advantages of this method are the ability to monitor the deformation of very complex shaped areas and the determination and real-time evaluation of the required quantities (displacement, strain, velocity, acceleration, or even stress [14]). Some mechanical properties, such as Young’s modulus and Poisson’s ratio, are also measured by this non-contact method [22]. The MERCURY RT system, provided by Sobriety company (Kuřim, Czech Republic), was used for all DIC measurements. This software was also useful for configuration and calibration of cameras. The optical probe was virtually created on the specimen before starting the measurements. This probe had to be aligned for both cameras. The optical probe provided an initial length and had to be visible during the whole mechanical test.

The study was supplemented with investigation of surfaces via scanning electron microscopy (SEM, Tescan Orsay Holding a.s., Brno, Czech Republic), and fractography was also performed. Preparations of the samples for the analyses were performed via ultrasound cleaning. The structure investigations were carried out using a Tescan Lyra 3 FIB/SEM microscope. The images were taken with the accelerating voltage of 10 kV.

## 3. FE Modelling

Since additive manufacturing technology is becoming increasingly popular, it is important to examine if the FEM can sufficiently predict accurate results with respect to the experimental response of materials. A number of analyses were thus performed in this study to validate the finite element model response under several loading conditions. Validation of the FEM was performed in terms of the comparison of results of the FEM with results from experiments.

**Figure 3:**
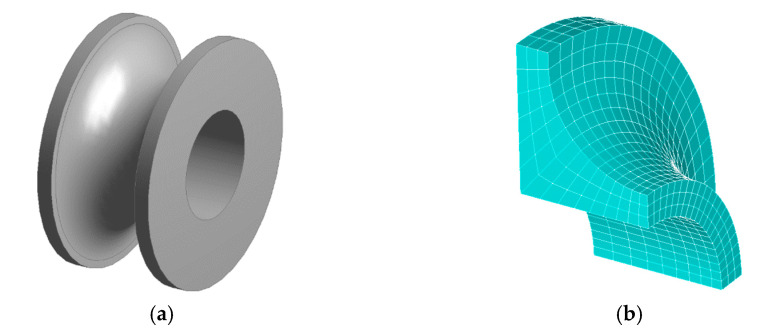
(**a**) Model of full specimen *A* cut-out, and (**b**) meshed specimen *A* utilizing 1/8 symmetry.

For the purposes of the FEM, the specimens were modelled as cut-outs of length equal to the initial length measured by the probe during experiments. Various levels of symmetry were utilized to reduce the computation time. For tensile analyses, 1/8 symmetry was used, and for torsion analyses, half symmetry was used. Models were meshed using linear hexagonal elements (SOLID185). Usage of mapped mesh and sizing settings ensured a regular and sufficiently-sized mesh to capture stress and strain gradients accurately (an example of mesh is in Figure 3b). All analyses were prescribed in the form of macros in Ansys Parametric Design Language (APDL) for easy, fast running, and automatic postprocessing of desired results.

**Figure 4:**
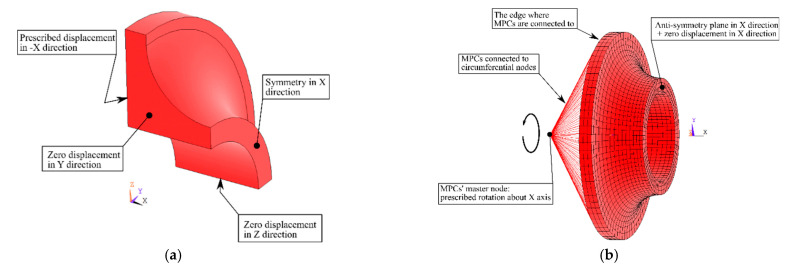
Description of boundary conditions for tensile load simulation (**a**) and for torsion load simulation (**b**) in simulations on specimen *A*.

Boundary conditions were set in accordance with the experimental loading conditions and symmetry assumptions. For the simulation of the tensile test, nodal displacement and symmetry plane boundary conditions were used. Those were applied on nodes within appropriate faces. See Figure 4a for an example of boundary conditions for tensile test simulations as it is similar for all specimens.

For the simulation of the torsion test of the specimen *A*, structural multipoint constraints (MPC184) were utilized to load the specimen in torsion. The nodal displacement in “x” direction and antisymmetry plane in “x” direction were used (see Figure 4b). Fix of displacement of nodes in “x” direction was sufficient since Ansys’s antisymmetry plane formulation treats, in this case, all other degrees of freedom. Observing the boundary conditions, one can see that all simulations were displacement-controlled.

To simulate the material response of specimens under conditions of tension and torsion, a suitable material model was needed. The chosen material model consists of nonlinear isotropic hardening, together with either Hill yield criterion or von Mises yield criterion. The linear elastic part of the model obeys Hooke’s law for three-dimensional problem

(1) $$\begin{array}{c} \varepsilon_{x}=\frac{1}{E}\left[\sigma_{x}-\mu \left(\sigma_{y}+\sigma_{z}\right)\right],{\;\varepsilon }_{y}=\frac{1}{E}\left[\sigma_{y}-\mu \left(\sigma_{x}+\sigma_{z}\right)\right],{\;\varepsilon }_{z}=\frac{1}{E}\left[\sigma_{z}-\mu \left(\sigma_{y}+\sigma_{x}\right)\right]\\ \gamma_{\mathrm{xy}}=\frac{\tau_{\mathrm{xy}}}{G},\;\gamma_{\mathrm{yz}}=\frac{\tau_{\mathrm{yz}}}{G},\;\gamma_{\mathrm{xz}}=\frac{\tau_{\mathrm{xz}}}{G} \end{array}$$

where $\sigma_{i}$ are the normal stress components, $\varepsilon_{i}$ are the normal strain components, $\tau_{\mathrm{ij}}$ are the shear stress components, $\gamma_{\mathrm{ij}}$ are the shear strain components, and G is the shear modulus. Thus, elasticity requires two input parameters of Young’s modulus *E* and Poisson ration μ. Values of the parameters are denoted in Table 2.

Isotropic hardening was suitable for this study since the loading was monotonic. Isotropic hardening during plastic deformation caused a uniform increase of the yield surface. This resulted in increased yield stress. Thus, the yield condition took the form:

(2) f(σ)−Y=0

where *f*(**σ**) is a function of the stress tensor **σ** and Y is the yield stress defining the current size of the yield surface. For the description of isotropic hardening, the Voce law was used. However, Voce law is a combination of linear and nonlinear isotropic variables and has the form:

(3) $$Y=\sigma_{Y}+R$$

where σ_Y_ is the initial yield stress and *R* is a new internal variable. The evolution of *R* is done by superposition of two parts:

(4) $$dR=dR_{1}+dR_{2},\;\;dR_{1}=R_{0}dp,\;\;dR_{2}=b{\left(R_{\infty }-R\right)}_{2}dp$$

where *R*_0_ and *R*_∞_ are material parameters and dp is the increment of accumulated plastic strain.

By integration of Equation (4), with zero initial values of p, $R_{1}$, and $R_{2}$, respectively, and use of Equation (3), the constitutive equation is obtained:

(5) $$Y=\sigma_{Y}+R_{0}p+R_{\infty }\left(1-e^{-\mathrm{bp}}\right).$$

The meaning of the material parameters is as follows: $R_{0}$ is the slope of the saturation stress, $R_{\infty }$ is the difference between the saturation stress and the initial yield stress, and b is the hardening parameter that governs the rate of saturation of the exponential term. Values of the parameters were optimized by the nonlinear least square method from the tensile test on the specimens printed in the vertical direction and are denoted in Table 3.

Hill yield criterion and von Mises criterion were used in this material model to compare their accuracy with respect to real additive manufactured specimen responses. Hill criterion was anisotropic, independent of hydrostatic pressure, and depended on the orientation of the stress relative to the axis of anisotropy, thus suitable for materials in which the microstructure influences the macroscopic behavior of the material, which is the case for additive manufactured steels [23,24]. Hill yield criterion was, in this study, utilized for the modelling of yield strength anisotropy based on build direction [25,26,27,28]. Hill yield criterion’s stress function for an ideally plastic material has the form:

(6) $$f\left(\sigma \right)\equiv F{\left(\sigma_{22}-\sigma_{33}\right)}^{2}+G{\left(\sigma_{33}-\sigma_{11}\right)}^{2}+H{\left(\sigma_{11}-\sigma_{22}\right)}^{2}+2L\sigma_{23}^{2}+2M\sigma_{31}^{2}+2N\sigma_{12}^{2}=\sigma_{y}^{2}$$

where *F*, *G*, *H*, *L*, *M*, and *N* are auxiliary coefficients, which are functions of the ratio of the scalar yield stress parameter $\sigma_{Y}$ and the yield stress in each of the six stress components. The relationships between coefficients and ratios are as follows [29,30,31]

(7) $$\begin{array}{c} F=\frac{1}{2}\left(\frac{1}{R_{22}^{2}}+\frac{1}{R_{33}^{2}}-\frac{1}{R_{11}^{2}}\right),\;G=\frac{1}{2}\left(\frac{1}{R_{33}^{2}}+\frac{1}{R_{11}^{2}}-\frac{1}{R_{22}^{2}}\right),\;H=\frac{1}{2}\left(\frac{1}{R_{11}^{2}}+\frac{1}{R_{22}^{2}}-\frac{1}{R_{33}^{2}}\right)\\ L=\frac{3}{2}\left(\frac{1}{R_{23}^{2}}\right),\;M=\frac{3}{2}\left(\frac{1}{R_{13}^{2}}\right),\;N=\frac{3}{2}\left(\frac{1}{R_{12}^{2}}\right),\\ R_{11}=\frac{\sigma_{11}^{y}}{\sigma_{y}},R_{22}=\frac{\sigma_{22}^{y}}{\sigma_{y}},R_{33}=\frac{\sigma_{33}^{y}}{\sigma_{y}},\\ R_{12}=\sqrt{3}\frac{\sigma_{12}^{y}}{\sigma_{y}},\;R_{23}=\sqrt{3}\frac{\sigma_{23}^{y}}{\sigma_{y}},\;R_{13}=\sqrt{3}\frac{\sigma_{13}^{y}}{\sigma_{y}} \end{array}$$

where the directional yield stress ratios $R_{\mathrm{ii}}$ and $R_{\mathrm{ij}}$ are related to the isotropic yield stress parameter $\sigma_{y}$, and $\sigma_{\mathrm{ij}}^{y}$ is the yield stress in the direction given by the value of subscripts *i* and *j*. Almost all directional yield stress ratios for uniaxial and torsional loading were equal to 1. The only different directional yield stress ratio was the directional ratio $R_{33}$, which was equal to 0.87. The ratio $R_{33}$ corresponded to the axial direction of all vertically printed specimens, thus introducing the effect of building direction into the material model.

The Von Mises yield criterion is isotropic, independent of hydrostatic pressure, and commonly used for metals, polymers, etc. In this study, accuracy of the von Mises yield criterion was examined by comparing with the real material response. The Von Mises stress function takes the form:

(8) $$f\left(\sigma \right)=\sqrt{\frac{{\left(\sigma_{1}-\sigma_{2}\right)}^{2}+{\left(\sigma_{2}-\sigma_{3}\right)}^{2}+{\left(\sigma_{1}-\sigma_{3}\right)}^{2}}{2}}$$

where σ_i_ are principal stresses.

## 4. Results

**Figure 5:**
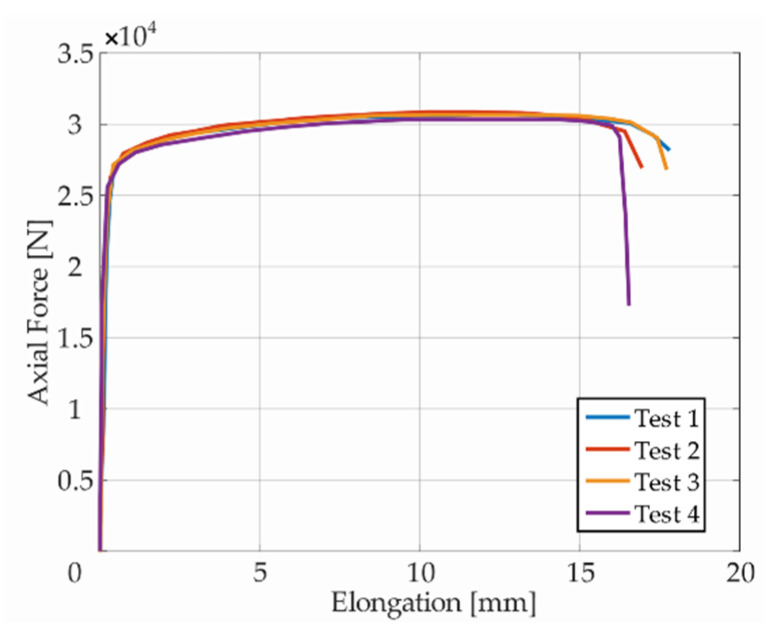
Dependency of force on elongation from tensile test of vertically printed specimens *T*.

All specimens were subjected to loading, as described above. Each test was deformation-controlled. Values of applied force or torque were recorded by the testing machine, and the values of deformation were recorded by the DIC system. Because the experimentally measured data embodies natural oscillations, the presented force vs. elongation diagrams were smoothed using functions of the Curve Fitting Toolbox in Matlab; see Figure 5 showing an example from monotonic tensile tests. The combination of moving average and smoothing splines led to sufficient results. Optimal smoothing parameters were chosen with respect to the size and character of the data sets.

**Figure 6:**
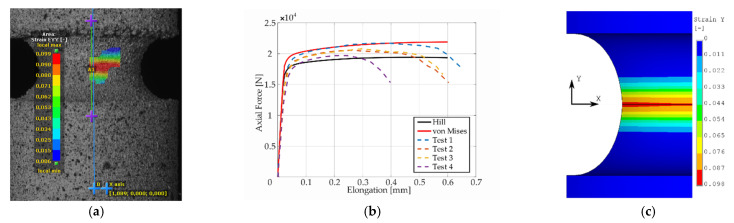
Longitudinal strain distribution from the digital image correlation (DIC) measurement (**a**), dependency of force on elongation compared to experimental and simulation results (**b**), and finite element method (FEM) result with Hill’s criterion material model (**c**) for specimen *A*.

**Figure 7:**
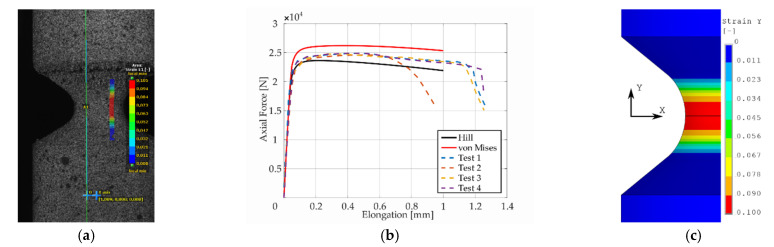
Maximal principal strain distribution (**a**), dependency of force on elongation compared to experimental and simulation results (**b**), and FEM result with Hill’s criterion material model (**c**) for specimen *B*.

**Figure 8:**
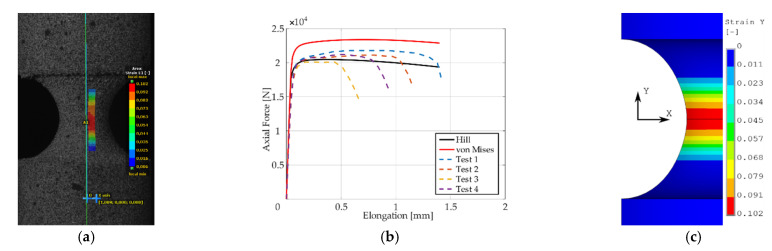
Maximal principal strain distribution (**a**), dependency of force on elongation compared to experimental and simulation results (**b**), and FEM result with Hill’s criterion material model (**c**) for specimen *C*.

**Figure 9:**
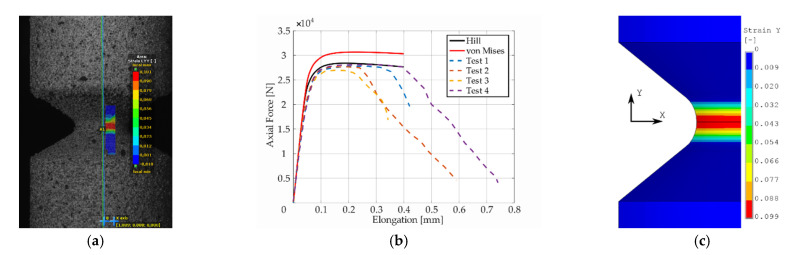
Longitudinal strain distribution (**a**), dependency of force on elongation compared to experimental and simulation results (**b**), and FEM result with Hill’s criterion material model (**c**) for specimen *D*.

**Figure 10:**
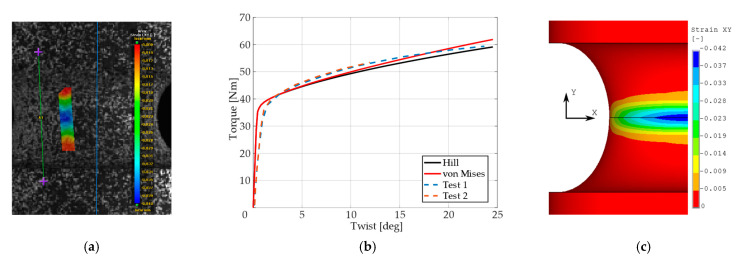
Shear strain distribution for torsional test (**a**), dependency of torque on twist compared to experimental and simulation results (**b**), and FEM result with Hill’s criterion material model (**c**) for specimen *A*.

Presentation of the simulation results and their comparison with respect to the experimental response of the “as printed” specimens follow next. The comparison was performed in the form of plots comprised of experimental smoothed responses in dashed lines and simulation results in full lines (see Figure 6, Figure 7, Figure 8, Figure 9 and Figure 10). Contours obtained by DIC measurements and FEM simulations were also compared for each specimen. A comparison of DIC and FEM contours served as the retrospective control of the simulation results with the calibrated material model.

**Figure 11:**
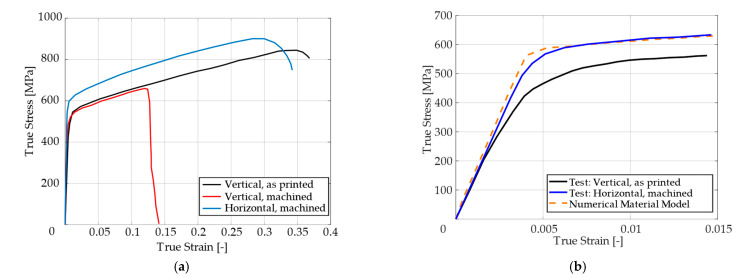
Three characteristic true stress–true strain curves for stainless steel 316L specimens produced by selective laser melting technology (**a**), and a detail of the material model response and experiment for the specimen “as printed” in the vertical direction and machined in the horizontal direction (**b**).

Ductility was calculated from the tensile test results for the specimen without a notch of T-type, considering

(9) $$\delta =\frac{L_{u}-L_{0}}{L_{0}}\times 100\%$$

where $L_{u}$ is the final length of the specimen after the test and $L_{0}$ is the initial gauge length of the testing part of the specimen. Initial gauge length for each specimen was 40 mm, and the elongations are presented in Figure 5. Specimens printed in the vertical direction without the machined outer surface had ductility from 42% to 45%. “As printed” specimens showed approximately 2/3 higher ductility than machined specimens printed in the vertical direction. The machined specimens had ductility 13–15%. The cutting velocity for the machined specimens was 60 m·min^−1^, the feed was 0.25 mm rev^−1^, the depth of cut was 1 mm, and they had roughness of Ra 0.8. This difference is also evident in Figure 11a, where the true stress–true strain curves for the three types of specimens are compared. The printed SS316L revealed surprisingly good ductility even when printed in the vertical direction (43% in comparison with 60% of the conventional SS316L), but just for the “as printed” variant.

The dependency of force on elongation was compared with machined specimens, which were printed in the vertical and horizontal directions. Vertically printed specimens had approximately the same yield stress, regardless of whether they were machined or “as printed”, as can be seen in Figure 11a. Thus, the machining used did not influence the microstructure.

However, the ductility differed significantly, as described above. It is also clear from Figure 11a that the vertically printed specimens had a lower yield stress than the horizontally printed specimens. This difference was approximately 75 MPa. This difference is evident from Figure 11b, where a detail of a vertical “as printed” and horizontal machined specimen is shown. The material model was determined for a vertical “as printed” specimen, and after adding just 75 MPa, the material model corresponded to the machined specimen printed in the horizontal direction. It is clearly shown that the machining, which was set in a manner to not affect stress-strain behavior of the material, can still lead to the reduction of material ductility.

**Figure 12:**
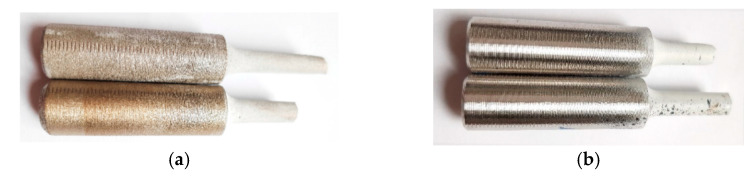
Fractured tensile test specimens: “as printed” (**a**); machined (**b**).

**Figure 13:**
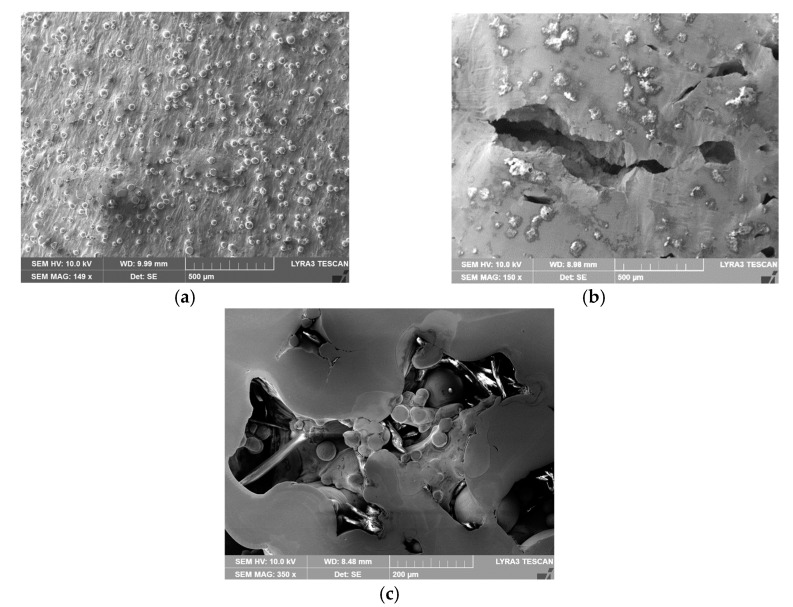
Surfaces of tensile test specimens: “as printed” (**a**), machined (**b**), and detail of surface of vertically printed material, i.e., original material for tensile test specimens (**c**).

The experimental work also included detailed microscopic observations of both the “as printed” and machined tensile test specimens. The analyses were performed on the surfaces of both the tensile specimens of type *T*, as well as at the locations of fractures. The fractured “as printed” and machined tensile specimens are depicted in Figure 12a,b, respectively. The figures clearly show that the surfaces of both specimens exhibited macroscopic differences, as the “as printed” specimen had a more or less silky smooth surface, whereas the machined specimen exhibited a shiny metallic surface. Figure 13a,b depict detailed images of the surfaces of both the “as printed” and machined specimens, respectively (acquired from the working part of the specimens featuring the smaller diameter). As can be seen, the “as printed” specimen featured a compact surface but with visible bumps (Figure 13a). Subsequent machining disturbed the surface and uncovered the voids beyond the bumps (Figure 13b). These features are supposed to originate from the 3D printing process. A detail of the original vertically-printed material, from which the specimens were fabricated, is depicted in Figure 13c.

**Figure 14:**
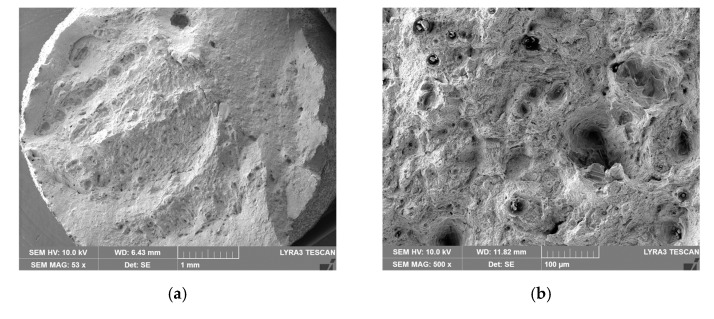

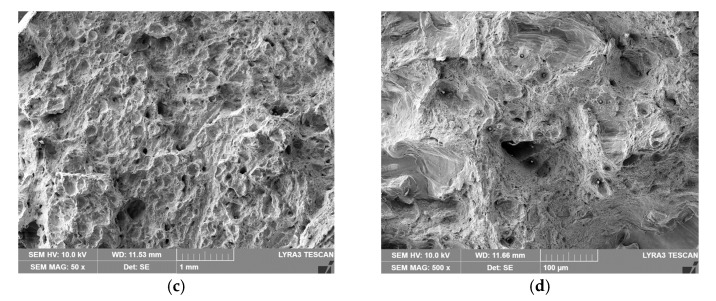
Details of fracture surfaces: “as printed”: 53× magnified (**a**), “as printed”: 500× magnified (**b**); machined: 50× magnified (**c**), and machined: 500× magnified (**d**).

Detailed images of the fractures of the “as printed” and machined specimens are depicted in Figure 14a–d. Mutual comparison of both fracture surfaces reveals that the characters of both fractures were different. The “as printed” specimen exhibited the character of ductile fracture (Figure 14a) but also the presence of local unmelted powder particles (Figure 14b). The machined specimen exhibited only the local occurrence of ductile fracture and prevailing features of brittle fracture (Figure 14c); the cracking seemed to have occurred along the boundaries of the powder particles, i.e., inter-granular fracture occurred (Figure 14d). The specimen also featured a frequent occurrence of unmelted powder particles, especially in the dimples, which originated from tensile loading during mechanical testing. Together with the surface cracks introduced by previous machining, these phenomena most probably contributed to the decreased strength and ductility of this specimen.

## 5. Discussion

During the test, when specimens with different notches were subjected to monotonic loading, it was evident that all specimens were stable up to the ultimate strength and showed very similar dependence of force (torque) on elongation (twist) for each notch type. Significant differences were observed in material fractures. This effect was attributed to the fact that the specimens were left in the “as printed” state. During production by the SLM method, inhomogeneity and porosity arose especially in the notches. This inhomogeneity and porosity can act as an indicator of material fracture. Porosity can affect the mechanical properties because these are cavities that lead to the fracture of the material. Comparison of the experimental and FEM results revealed the following: observing the responses under tension for specimen *A* (Figure 6), one could see that the numerical model using the Hill’s yield criterion (further FEM-Hill) described the experiment with the lowest ductility and axial force better. On the contrary, the numerical model using the von Mises yield criterion (further FEM-Mises) described the experiment with the highest ductility and axial force better. Thus, for this specimen, it cannot be unequivocally said which material model describes the experiment better. Next, observing the responses under tension for specimens *B* and *C* (Figure 7 and Figure 8), one could see that the FEM-Hill described the yield region better than FEM-Mises. Nevertheless, after reaching the axial force ‘plateau’, the experimental responses were generally in between the two numerical model responses, with FEM-Hill rather underestimating and FEM-Mises overestimating the axial force. To conclude the insights, it can be said that FEM-Hill described the experimental responses better in this case, since it accurately captured the beginning of the nonlinear response (which corresponded to the yield region) compared to FEM-Mises. Next, observing the responses under tension for specimen *D* (Figure 9), one could see that the FEM-Hill described the experimental response generally better during the whole examined elongation range. In this case, the FEM-Mises clearly overestimated the axial force. Finally, observing the responses under torsion for specimen *A* (Figure 10), one could see that the FEM-Hill and FEM-Mises responses were identical up to the twist angle of about 10 degrees and then a minor difference could be observed. Both numerical models then described the experimental response well, mainly in the plastic region.

The experiments also showed a significant difference in ductility between specimens that were left in the “as printed” state and specimens that were machined. Ductility was evaluated on specimens without notches. The machined specimens had a prescribed roughness of Ra 0.8. It should be noted that both types of specimens were printed in the vertical direction. The primary idea of why the machined specimens had a 2/3 lower ductility was that the layers that were laid during 3D printing (and the pores were primary located in them) were parallel to the machine knife during machining. As a result of machining, the partial segments broke out more easily and microscopic cracks appeared on the surface of the material with the occurrence of pores (stress concentration). This lead to an earlier fracture. This effect could also be observed during the microscopic analysis of both types of specimens in this study. On the specimens that were produced by printing in the horizontal direction, the machine knife then acted perpendicular to the laid layer during machining and therefore the microscopic segments did not have to be so easily broken out.

## 6. Conclusions

In this study, four types of notched specimens were investigated under monotonic testing. Loading path was pure tension and pure torsion. In addition, the specimen without notches was investigated by a tensile test. The specimens were made from stainless steel 316L, produced by selective laser melting technology in the “as printed” state. A DIC measurement was used to gather data during testing and for postprocessing.

The main insights can be listed as:

- Specimens “as printed” prepared in the vertical direction had good ductility; only about 15% lower than specimens produced by conventional methods.
- Machining had a negative effect on ductility if the specimen was loaded in the printing direction. “As printed” specimens showed approximately 2/3 higher ductility than machined specimens.
- Additionally, the fracture was different for “as printed” and machined specimens. While “as printed” specimens had the character of ductile fracture, the machined specimen only had the local occurrence of ductile fracture and the prevailing features of brittle fracture.
- Vertically printed specimens had lower yield stress than horizontally printed specimens, approximately by 75 MPa, regardless of whether they were in the “as printed” state or machined.
- The FEM-Mises generally overestimated the axial force, leading to a stiffer response under tensile loading.
- The FEM-Hill showed the ability to better describe the yield range and accurately capture (or slightly underestimate) the axial force within the force ‘plateau’ range.
- The results confirmed the suitability of the material model with Hill’s yield criterion to adequately describe the SLM produced material response under tension as the FEM-Hill (which included the yield stress reduction in the printing direction) better captured the yield region than FEM-Mises (which behaved isotropically).

Regarding the comparison of experimental and FEM analysis results under torsion, it was found that the FEM-Mises and FEM-Hill behaved basically identically within the whole examined twist range, capturing the experimental response well, mainly in the nonlinear part. This implies that the reduction of tensile yield strength in the printing direction in the FEM-Hill did not have the influence on the material response under torsional loading in this case. Nevertheless, further studies are needed to fully capture, understand, and address the torsional behavior and printing direction influence on the response during the torsional loading. The nonlinear isotropic hardening model with Hill yield condition gives acceptable results under given multiaxial stress states. Under monotonic loading, the nonlinear isotropic hardening model is equivalent to Chaboche kinematic hardening model with two back-stress parts when the second back-stress part is linear. Therefore, a good correlation can also be expected for the Chaboche model (in monotonic loading cases).

It should also be noted that the vertically printed specimens used in this study were not heat treated in any way, such as annealing to remove internal stress. This can also affect the results, of course, especially in the case of horizontally printed specimens. However, to maintain a high yield stress of the material, it is advisable to choose a gentle heat treatment that preserves the fine-grained microstructure of the material. The following study deals with the comparison of experiments on notched specimens in the “as printed” state and is finally modified by machining. Attention is paid to the adherence of the specimen geometry and surface roughness [32]. In the field of numerical modeling, numerical procedures capturing ductile failure on “as printed” specimens and comparison with conventionally produced specimens with the inclusion of small punch tests are used [33].

## Figures and Tables

**Table 1:** 3D printing parameters.

3D Printer:	Renishaw AM400
Powder description:	SS Powder AISI 316L (DIN 1.4404)
Powder Particle Size:	15–45 µm
Layer Thickness:	50 µm
Focus Size:	70 µm
Print Strategies:	Chessboard
Border Power:	110 W
Border Exposure Time:	100 µs
Border Point Distance:	20 µm
Hatches Power:	200 W
Hatches Exposure Time:	80 µs
Hatches Point Distance:	60 µm
Jump speed:	5000 mm·s^−1^
Dosing time:	7 s
Melting range:	1371 to 1399 °C
Concentration of Oxygen:	<0.1% O_2_
Inert Gas:	Argon
Purity:	5.0 (99.998%)

**Table 2:** Elastic parameters.

Parameter	Value	Unit
*E*	183	GPa
Μ	0.3	-

**Table 3:** Voce law parameters.

Parameter	Value	Unit
$\sigma_{Y}$	575	MPa
$R_{0}$	950	MPa
$R_{\infty }$	60	MPa
b	125	-

## Data Availability

The data presented in this study are available on request from the corresponding author. The data are not publicly available due to: All data are presented in the form of graphs in this article.
